# General practitioners’ capability, opportunity and motivation to discuss physical activity at the 6-8 week postnatal check in the UK: a qualitative study using the COM-B model

**DOI:** 10.1136/bmjopen-2026-122011

**Published:** 2026-10-23

**Authors:** Joseph Morwood, Peter C Rouse, Marlize De Vivo

**Affiliations:** 1 University of Bath, Bath, UK; 2 Advanced Wellbeing and Research Centre, Sheffield Hallam University, Sheffield, UK

**Keywords:** Postpartum Period, General Practice, Health Education

## Abstract

**Abstract:**

**Objectives:**

To explore general practitioners’ (GPs) perceived capability, opportunity and motivation to discuss physical activity during the 6-8 week postnatal check, using the COM-B model of behaviour change as conceptual framework.

**Design:**

Qualitative study using semi-structured interviews, with data analysed thematically using the COM-B (capability, opportunity, motivation and behaviour) model as the conceptual framework.

**Setting:**

UK National Health Service (NHS) general practice.

**Participants:**

Seven practising NHS GPs who had conducted at least one 6-8 week postnatal check within the preceding 12 months.

**Results:**

Seven themes were derived from the interviews conducted. Maternal history, GP pregnancy experience and access to information resources were those found relevant to GPs’ capability to discuss physical activity. Patients’ social background and GPs’ clinical priorities were identified as the main barriers to opportunity. The balance between GPs’ positive outlooks on physical activity and maternal readiness to assimilate advice was described in relation to motivation to discussion.

**Conclusions:**

Although GPs are motivated to promote postnatal physical activity, structural and contextual constraints limit consistent delivery. Improving access to efficient, credible signposting resources may enhance capability and support sensitive integration of physical activity promotion within routine care.

**STRENGTHS AND LIMITATIONS OF THIS STUDY**

- Applies the COM-B (capability, opportunity, motivation and behaviour) model to explore factors influencing physical activity discussions within the 6– 8-week postnatal consultation.
- Provides qualitative insight into general practitioners’ (GPs) experiences of discussing physical activity during routine postnatal care.
- The researcher’s position as a practising GP was explicitly considered in relation to data collection and interpretation.
- The small, self-selecting sample may overrepresent GPs with an interest in physical activity or health promotion.
- Coding and interpretation were undertaken by a single researcher, limiting the range of analytic perspectives represented.

## Introduction

 The postnatal period represents an intergenerational link for health protective behaviours such as physical activity. Mothers who display higher physical activity levels raise more physically active children^1 2^ and active children are more likely to become active adults.^3^ Both children and adults gain significant reductions in all-cause mortality by engaging in sufficient physical activity throughout life.^4^ For this reason, minimum amounts are recommended in the Chief Medical Officer’s physical activity guidelines for all groups including pregnancy. It is suggested that following pregnancy, adults reintroduce and build back to the same volume of physical activity as before pregnancy, aiming for 150 min of moderate activity per week.^5^

However, evidence suggests that moderate- and vigorous-intensity activity decreases as pregnancy progresses, rarely returning to pre-pregnancy levels, placing mothers at risk of continued inactivity.^6 7^ There are multiple barriers to women engaging in physical activity antenatally and postnatally^8 9^; however, lack of information and encouragement ranks highest in barriers pertaining to the healthcare environment.^10^ Women provided with postnatal activity guidance are more likely to return to activity^11^ and physicians rank highest in women’s reporting of influence on beliefs about physical activity and pregnancy.^12^ Pregnancy has been identified as a ‘teachable moment’ that provides a cue to better potentiate healthy lifestyle changes including physical activity.^13^

In England, women are offered a postnatal consultation with a general practitioner (GP) between 6 and 8 weeks after birth. Provision of this consultation has formed part of the GP contractual framework since 2020. NHS England guidance recommends a holistic assessment of maternal physical and mental health and identifies physical activity as an area for health promotion during the consultation. The 6-8 week postnatal check therefore provides an opportunity for GPs to discuss physical activity following childbirth. For this reason, ‘Returning to physical activity’ has been highlighted as a topic of inclusion within ‘What good looks like’ NHS England 6–8 week check guidelines.^14^

However, this opportunity to discuss physical activity currently appears under-utilised in NHS General Practice.^15 16^ As a result, clinicians’ beliefs about their role in postnatal physical activity promotion have been identified as a research priority.^17^

With NHS Primary Care facing recurrent crises,^18^ understanding what shortfalls reflect practitioner deficits and which reflect systemic barriers is complex to assess. Moreover, challenges to GPs cross multiple domains, not only related to environment and resources but also emotional and physical restraints.^18^ More could be understood about healthcare professionals’ experience in providing this advice to contextualise outcomes. De Vivo and Mills sought this qualitative insight among NHS Midwives whose perspectives on providing physical activity advice not only highlighted institutional barriers in lack of resources and training but also generated focus on opportunities to discuss physical activity.^19^

Interventions for enhancing physical activity delivery have been designed successfully using the Behaviour Change Wheel framework.^20^ This framework relies on the requisite conditions of capability, opportunity and motivation, for which a single behaviour change requires modification of at least one (otherwise known as the ‘COM-B’ approach).^21^ The COM-B model has been successfully used to guide exploration of themes around physical activity in pregnancy using focus groups.^22^ Understanding the capabilities, opportunities and motivation of GPs to discuss physical activity at the 6-8 week postnatal check can be a useful framework to identify opportunities for future intervention. As a result, the following research questions were developed:

- What are GPs’ perceived capabilities to provide physical activity advice and guidance at the 6–8 week postnatal check?
- What are GPs’ perceived opportunities to provide physical activity advice and guidance at the 6–8 week postnatal check?
- What are GPs’ perceived motivations to provide physical activity advice and guidance at the 6–8 week postnatal check?

## Methods

### Design

A qualitative study using semi-structured interviews. Data were analysed thematically, with the COM-B model providing the conceptual framework.

### Participants and recruitment

Practising NHS GPs were recruited via professional networks and social media. Eligible participants had delivered at least one 6-8 week postnatal check within the previous 12 months and had membership with the Royal College of General Practitioners with a licence to practise in the UK. Participants were provided with a participant information sheet, and informed consent was obtained via an electronic consent form before the interview. GPs were not compensated for their participation.

### Data collection

Interviews were conducted via Zoom between November 2024 and April 2025 by the first author (JM), a practising NHS GP. An initial pilot interview with an NHS GP informed development of the semi-structured topic guide, which was structured around the COM-B domains and refined following a second pilot interview (online supplemental A). Sessions lasted 40-65 min, were audio-recorded and transcribed using Zoom software and then were anonymised and checked against the recordings for accuracy. Transcripts were not returned to participants for comment or correction, and no repeat interviews were conducted.

### Analysis

Data were analysed thematically using a combination of deductive and inductive coding. The COM-B model provided the conceptual framework, with capability, opportunity and motivation used a priori to structure the deductive analysis. Inductive coding was also used to identify additional patterns within participants’ accounts. Following familiarisation with the transcripts, the first author (JM) undertook line-by-line coding using Microsoft Excel. Codes were reviewed iteratively and grouped into themes reflecting participants’ experiences of discussing physical activity during the 6-8 week postnatal consultation.

Within COM-B, the three overarching components are further specified as physical and psychological capability, physical and social opportunity and reflective and automatic motivation. These six subcomponents informed the coding process, but the final themes were organised within the three overarching components. This approach reflected the study aim of identifying broader behavioural determinants influencing GPs’ discussions of physical activity and accommodated areas of overlap across the COM-B subcomponents. All transcripts were coded and analysed by the first author using Microsoft Excel. The supervisory team provided methodological guidance and contributed to reflexive discussion of the developing analysis but did not independently code the data. Sample size was informed by the concept of information power.^23^ The study addressed a focused research aim using a professionally homogeneous sample of GPs with direct experience of conducting the 6-8 week postnatal consultation in the UK. Interview data were reviewed throughout data collection to consider the richness and relevance of the information generated, and recruitment concluded when the dataset was judged to provide sufficient information to address the study aim.

### Researcher reflexivity

The interviews, transcription and analysis were undertaken by the first author (JM) as part of an MSc research project. JM has a professional interest in primary care and physical activity promotion, which informed the choice of research topic and facilitated an understanding of the clinical context. Recognising that prior knowledge and experiences could influence data collection and interpretation, a semi-structured interview guide was used to promote consistency across interviews. Throughout analysis, coding decisions and interpretations were continually reviewed against the original data to ensure that themes accurately reflected participants’ accounts.

### Patient and public involvement

Patients and the public were not involved in the design, conduct or reporting of this study.

The Standards for Reporting Qualitative Research (SRQR) reporting guideline^24^ was used to draft this manuscript, and the SRQR reporting checklist^25^ was used when editing, included in online supplemental B.

## Results

Of the 12 GPs who initially consented to participate, seven completed an interview within the study period. Six participants were female and one was male, and four had previously experienced pregnancy. Participants were aged 32-56 years, with a mean age of 40.86 years (SD 8.49). Two reported engaging in less than 90 min of physical activity per week, while five reported more than 150 min per week. Seven themes were identified across the COM-B domains: three related to capability, two to opportunity and two to motivation.

### Capability

#### Theme one: maternal history

##### Maternal history subtheme: clinical complexity

GPs consistently described variation in confidence depending on obstetric complexity. Advice for women following uncomplicated vaginal delivery was perceived as straightforward and within expected GP competence:

Your sort of general patient who does a bit of exercise… I would feel pretty confident. (GP1)

In contrast, more complex clinical scenarios, particularly caesarean sections, reduced confidence and introduced uncertainty around safety and appropriate progression:

Perhaps maybe less confident with patients after Caesarean section… you obviously don’t want to be advising people to do things that could cause injury and harm. (GP2)

Some participants described their advice in these contexts as vague or non-specific:

After the mum has had a C-section…If I’m being honest with you, my advice is pretty woolly. (GP6)

This uncertainty appeared linked to concern about causing harm, suggesting that capability was not solely knowledge-based but also risk-sensitive.

##### Maternal history subtheme: managing high baseline fitness and athletic return

A second dimension of capability is related to advising women with high levels of pre-pregnancy fitness. GPs expressed reduced confidence when counselling women aiming to return to higher-intensity or competitive activity:

If it was someone who was sort of an athlete… I would feel out of my depth. (GP1)

Similarly:

So, if you had somebody who was quite high level…who regularly did lots of exercise classes at the leisure centre…I don’t know when you can start doing that… I wouldn’t have a clue. (GP2)

In these cases, GPs often defaulted to conservative advice, sometimes emphasising caution over progression:

I’m kind of reining them in… just hold your horses a little bit. (GP3)

### Theme two: influence of personal pregnancy experience

Personal or vicarious experience of pregnancy emerged as a strong modifier of perceived capability. GPs who had experienced pregnancy themselves, or through close partners, described greater confidence and insight:

I’ve had three caesareans and so it’s been quite a long road myself, getting back to exercising safely… it’s probably made me more confident. (GP1)

With my wife having been through it, I kind of have so much more insight… it offers more confidence in delivering the advice. (GP4)

These experiences often informed more nuanced understanding of recovery trajectories and common challenges, particularly pelvic floor dysfunction and delayed return to exercise:

I returned to Netball, maybe after 8 months or something. I didn’t have any problems with pelvic floor that I thought until I started exercising…. So it’s an important conversion to have.(GP5)

### Theme three: access to information and signposting resources

Across participants, access to reliable and easily shareable resources was identified as a key facilitator of capability. GPs described a desire for structured, credible materials to support advice delivery:

If there was like, somewhere where women could access sort of exercise classes that were very specifically postnatal focused… that would be brilliant. (GP1)

Existing resources were used where available, but often described as limited or insufficiently specific:

I’m always directing people to NHS resources… but if there was something more specific…that would be helpful. (GP3)

Some GPs highlighted the value of signposting as an alternative to in-consultation advice, allowing patients to engage at their own pace:

I send the leaflet out… if it’s not the right time that’s cool as well. (GP5)

Digital formats, including apps and social media, were perceived as particularly useful, although identifying trustworthy sources was challenging:

Having resources that you can text people or signpost people to and therefore you can bring up the conversation…It’s actually really hard to find. I’m not having huge success. (GP4)

### Opportunity

#### Theme one: social and environmental context

Participants consistently described how patients’ social and environmental contexts influenced the feasibility of physical activity and therefore the relevance of discussion within consultations.

GPs working in more affluent areas described patients with greater access to structured exercise opportunities:

Most of them had already, you know, signed up with some postnatal yoga or Pilates instructor… they had the money to do all those classes privately. (GP1)

In contrast, those working in deprived or rural settings highlighted significant structural barriers:

Our patients are either experiencing homelessness or refugees… physical exercise is not a priority. (GP2)

There’s nowhere to walk… no footpaths… they’re kind of stuck in their houses. (GP3)

Financial constraints were also prominent:

I don’t have the money to pay for a gym. (GP3, describing patient experiences)

These contextual factors influenced whether physical activity discussions were perceived as appropriate in the absence of widening access to physical activity opportunities:

Lifestyle conversations are great, but they require agency… they can widen health inequalities… They should be delivered in the context of a system change. (GP4)

#### Theme two: competing clinical priorities and time constraints

Time pressure emerged as the most consistent barrier to opportunity. The 6–8 week check was described as a consultation with multiple competing demands:

There’s just so much that we’re trying to do. (GP2)

Participants emphasised that both maternal and infant needs required attention, often prioritising acute or complex issues over health promotion:

Your attention ends up on those things… then immediately the focus doesn’t seem to be then on health promotion. (GP3)

Even in uncomplicated consultations, time was perceived as insufficient:

Even if there’s no complicated issues… that usually takes 10 to 15 min. (GP5)

Discussing physical activity was therefore seen as requiring additional time, often at the cost of running late:

I’d love to have a couple of minutes conversation about exercise, diet and keeping yourself healthy. But actually that’s a choice of running late every time. (GP5)

As a result, physical activity advice was often omitted unless directly raised by the patient.

### Motivation

#### Theme one: positive beliefs about physical activity

All participants expressed strong belief in the benefits of physical activity, particularly in relation to maternal mental health and long-term family well-being:

You’re getting them when they’re still relatively young and hopefully being able to put in those positive lifestyle messages early on and prevent… a whole load of problems later on in life. (GP1)

We know that physical activity really helps with mental health… Getting that sort of set in your brain in that period also, then can influence the child. (GP3)

Some participants described physical activity promotion as particularly meaningful compared with other aspects of care:

You can make a real difference with it more than just writing a prescription. (GP7)

These beliefs contributed to generally high motivation across participants.

#### Theme two: maternal readiness and perceived sensitivity

Despite high motivation, participants frequently described moderating their behaviour based on perceived maternal readiness. The early postnatal period was seen as a time of vulnerability, with multiple competing priorities:

It can feel quite judgy… the last thing they want is someone telling them, ‘And now you need to go out and do some exercise.’ (GP1)

Sleep deprivation, feeding challenges and emotional adjustment were commonly cited:

If they’re battling with other things… it just might not be the right time to discuss it. (GP3)

Some women are just so knackered that there’s no point even mentioning it. (GP7)

Some GPs questioned whether the 6–8 week check was the appropriate timing altogether:

Often it’s just too early. I wish we could have another check… at about 6–8 months. (GP1)

Others described adapting their approach to reduce perceived pressure, for example, by providing written information rather than verbal advice:

I send the leaflet out and just say, you know this is the information here. If you want it, have a read. If it’s not the right time that’s cool as well. (GP5)

This balancing of motivation with sensitivity suggests that behaviour was shaped not only by belief in benefits but also by relational and contextual considerations.

## Discussion

This study explored GPs’ capability, opportunity and motivation to discuss physical activity during the 6-8 week postnatal check using the COM-B model as a conceptual framework. The findings demonstrate that while GPs are generally motivated and positively disposed towards promoting physical activity, opportunity constraints and contextual sensitivity significantly limit consistent delivery.

Capability was influenced by clinical complexity, prior experience and access to resources. Opportunity was most constrained by time pressure and competing clinical priorities within the consultation, as well as wider socioeconomic barriers affecting patients’ ability to engage with physical activity. Motivation was high, but frequently moderated by concerns about maternal readiness and the risk of appearing insensitive during the early postnatal period.

These findings align with previous research demonstrating that physical activity is inconsistently addressed in postnatal consultations. Macdonald *et al* reported that exercise is ‘rarely’ discussed during the 6-8 week check, alongside other lifestyle topics such as diet and smoking.^16^ The present study provides additional depth, suggesting that this omission is not due to lack of clinician motivation, but rather reflects structural and contextual constraints within the consultation.

Time pressure emerged as the dominant barrier to opportunity, consistent with wider evidence of increasing workload and consultation complexity in UK primary care.^26^ The 6-8 week check is unique in that it encompasses both maternal and infant assessment, often involving multiple competing priorities within a limited time frame.^16^ Participants described prioritising clinical uncertainty, such as infant abnormalities or maternal mental health concerns, over health promotion. This supports the interpretation that physical activity advice is deprioritised not due to perceived irrelevance, but due to triaging within constrained consultation time.

Capability-related findings also extend existing literature. While previous studies have identified lack of training as a barrier to physical activity discussions,^19^ this study highlights the situational nature of confidence. GPs felt capable in straightforward cases but less so in complex scenarios, particularly following caesarean sections or when advising individuals who were previously highly active. This suggests that capability is not uniformly deficient but instead varies depending on perceived clinical risk and scope of practice.

The influence of personal pregnancy experience is notable and consistent with literature demonstrating that clinicians’ personal health experiences can shape clinical practice.^27^ In this study, lived experience appeared to enhance both confidence and empathy, particularly in relation to pelvic floor dysfunction and realistic recovery timelines. This may help explain variation in practice beyond formal training and highlights the role of experiential knowledge in shaping behaviour.

The identification of resource availability as a facilitator aligns with behaviour change theory and prior intervention research.^20 21^ Participants frequently described signposting to digital or written resources as a practical strategy to overcome time constraints and support patient autonomy. This reflects broader trends in primary care towards digital communication and may represent a feasible intervention within existing system limitations.^28^

### Maternal readiness and relational considerations

An important finding of this study is the central role of maternal readiness in shaping GPs’ decisions about when and how to discuss physical activity. While GP motivation to promote physical activity was high, participants consistently described modifying their approach based on perceived patient receptiveness.

The early postnatal period was characterised as a time of significant physical, emotional and social adjustment, with competing priorities including sleep deprivation, infant feeding and mental health challenges. In this context, physical activity advice was sometimes perceived as potentially insensitive or poorly timed. This aligns with qualitative literature describing postnatal stressors and negative experiences of insensitive healthcare interactions.^9 29^

The concept of pregnancy and the postnatal period as a ‘teachable moment’ for behaviour change may therefore require refinement. While motivation for behaviour change may exist, the findings suggest that timing and delivery are critical and that receptiveness may vary considerably between individuals. These findings are consistent with established principles of patient-centred care, which emphasise understanding patient’s experiences, concerns and priorities before jointly agreeing management plans.^30 31^

### Health inequalities and structural constraints

Participants highlighted the influence of socioeconomic and environmental factors on both opportunity and relevance of physical activity discussions. Barriers such as lack of safe walking environments, financial constraints and limited access to facilities were commonly described.

These findings raise important concerns regarding the potential for physical activity promotion to widen health inequalities, particularly if interventions disproportionately benefit those with greater resources and agency. This reflects broader public health literature demonstrating that 6–8 week check delivery varies across socioeconomic groups^32 33^ and that lifestyle interventions may have unequal impacts across socioeconomic groups.^34 35^

The implication is that interventions targeting GP behaviour alone may be insufficient. Effective promotion of postnatal physical activity is likely to require system-level changes, including improved access to community resources, infrastructure and equitable service provision.

### Implications for practice and policy

The findings suggest several potential avenues for intervention. First, enhancing access to credible, easily shareable resources may improve GP capability and facilitate discussion within time-limited consultations. Digital tools, including text messaging links and curated online content, may be particularly valuable. Second, training and guidance may be most effective if focused on specific areas of uncertainty, such as return to activity following caesarean section or for highly active individuals, rather than general physical activity promotion. Third, consideration should be given to timing of interventions. While the 6-8 week check represents an important contact point, some participants questioned whether this is the optimal moment for behaviour change discussions. Additional or alternative follow-up opportunities may enhance effectiveness.

Finally, addressing structural barriers and inequalities will be essential to ensure that physical activity promotion is both feasible and equitable.

### Strengths and limitations

A key strength of this study is the use of the COM-B framework to provide a structured and theoretically informed analysis of GP behaviour. The use of in-depth qualitative interviews enabled exploration of contextual and experiential factors. Researcher reflexivity was considered throughout data collection and analysis, acknowledging the first author’s professional background as an NHS GP and its potential influence on data interpretation.

Several limitations should be acknowledged. The sample size was small and recruited through professional networks, which may have resulted in selection bias towards GPs with an interest in physical activity or lifestyle medicine. Self-reported behaviours may not have reflected actual clinical practice, and participants may have modified responses due to awareness of the study topic. Male GPs were under-represented, limiting exploration of potential gender differences in perspectives.

Coding and interpretation were undertaken by a single researcher and therefore reflect their situated analytic perspective. Reflexive discussion with the supervisory team was used throughout the analytic process to interrogate developing interpretations. The study did not involve postnatal women in its design; their involvement may have provided additional perspectives to inform development of the interview guide and interpretation of the findings.

## Conclusions

GPs are motivated to promote physical activity at the 6-8 week postnatal check and recognise its importance for maternal and child health. However, delivery is constrained by limited consultation time, competing clinical priorities and broader socioeconomic factors.

Capability varies depending on clinical complexity and experience, while motivation is moderated by sensitivity to maternal readiness in the early postnatal period. Interventions focused on improving access to credible resources and supporting flexible, patient-centred approaches may enhance integration of physical activity promotion within routine care.

## Supplementary material

10.1136/bmjopen-2026-122011 online supplemental file 1

10.1136/bmjopen-2026-122011 online supplemental file 2

## Data Availability

Data are available upon reasonable request.

## References

[1] Hesketh KR, Goodfellow L, Ekelund U (2014). Activity levels in mothers and their preschool children. Pediatrics.

[2] Hesketh KR, Brage S, Cooper C (2019). The association between maternal-child physical activity levels at the transition to formal schooling: cross-sectional study. Int J Behav Nutr Phys Act.

[3] Telama R, Yang X, Viikari J (2005). Physical activity from childhood to adulthood. Am J Prev Med.

[4] Lear SA (2017). The effect of physical activity on mortality and cardiovascular disease: the PURE study. Lancet.

[5] Department of Health and Social Care (2019). UK Chief Medical Officers' Physical Activity Guidelines. https://assets.publishing.service.gov.uk.

[6] Abbasi M, van den Akker O (2015). A systematic review of changes in women’s physical activity before and during pregnancy and the postnatal period. J Reprod Infant Psychol.

[7] Pereira MA, Rifas-Shiman SL, Kleinman KP (2007). Predictors of change in physical activity during and after pregnancy: Project Viva. Am J Prev Med.

[8] Evenson KR, Moos MK, Carrier K (2009). Perceived barriers to physical activity among pregnant women. Matern Child Health J.

[9] Ayers S, Crawley R, Webb R (2019). What are women stressed about after birth?. Birth.

[10] Edie R, Lacewell A, Streisel C (2021). Barriers to Exercise in Postpartum Women: A Mixed-Methods Systematic Review. J Womens Health Phys Therap.

[11] Donnelly GM, James M, Coltman C (2022). Running during pregnancy and postpartum: advice and guidance. J Womens Health Phys Ther.

[12] Krans EE, Gearhart JG, Dubbert PM (2005). Pregnant women’s beliefs and influences regarding exercise during pregnancy. J Miss State Med Assoc.

[13] Phelan S (2010). Pregnancy: a “teachable moment” for weight control and obesity prevention. Am J Obstet Gynecol.

[14] NHS England (2023). GP six to eight week maternal postnatal consultation – what good looks like guidance. https://www.england.nhs.uk.

[15] Active Pregnancy Foundation (2023). Overlooked & underserved: pregnant & postnatal women’s engagement, opportunities & resources for physical activity.

[16] Macdonald C, Cross-Sudworth F, Quinn L (2025). Content and timing of the 6–8 week maternal postnatal check: a mixed-methods study. BJGP Open.

[17] Reid H, Smith R, Calderwood C (2017). Physical activity and pregnancy: time for guidance in the UK. Br J Sports Med.

[18] Beech J, Fraser C, Gardner T (2023). Stressed and overworked: What the commonwealth fund’s 2022 survey means for the UK.

[19] Vivo M, Mills H (2019). Insights into midwives’ perspectives of providing physical activity advice. BMC Pregnancy Childbirth.

[20] Webb J, Foster J, Poulter E (2016). Increasing the frequency of physical activity very brief advice for cancer patients. Development of an intervention using the behaviour change wheel. Public Health.

[21] Michie S, van Stralen MM, West R (2011). The behaviour change wheel: a new method for characterising and designing behaviour change interventions. Implement Sci.

[22] Gilroy V, Vivo M, Lawson R (2019). Educational campaign to support pregnant women and new mothers. UKactive.

[23] Malterud K, Siersma VD, Guassora AD (2016). Sample Size in Qualitative Interview Studies: Guided by Information Power. Qual Health Res.

[24] O’Brien BC, Harris IB, Beckman TJ (2014). Standards for reporting qualitative research: A synthesis of recommendations. Academic Medicine [Internet].

[25] O’Brien BC, Harris IB, Beckman TJ, Harwood J, Albury C, Beyer JD (2025). The EQUATOR network reporting guideline platform.

[26] Calderón-Larrañaga A (2024). Trends in clinical workload in UK primary care 2005–2019. Br J Gen Pract.

[27] Fox F, Harris M, Taylor G (2009). What happens when doctors are patients. Br J Gen Pract.

[28] Leahy D, Lyons A, Dahm M (2017). Use of text messaging in general practice. Br J Gen Pract.

[29] Campbell D (2025). Exercise and screen time advice for new mothers angers parenting groups. The Guardian.

[30] Stewart M, Brown JB, Weston WW (2014). Patient-centered medicine: Transforming the clinical method.

[31] Carroll JK, Antognoli E, Flocke SA (2011). Evaluation of physical activity counseling in primary care using direct observation of the 5As. Ann Fam Med.

[32] Cameron EE, Sedgwick R, McConnachie A (2020). Outcomes of postnatal checks for mothers in primary care. BMJ Open.

[33] Raleigh VS, Hussey D, Seccombe I (2010). Ethnic and social inequalities in maternity care. J R Soc Med.

[34] Lorenc T, Petticrew M, Welch V (2013). What types of interventions generate inequalities. J Epidemiol Community Health.

[35] Morgan K, Rahman M, Moore G (2020). Patterning in patient referral to exercise schemes. Int J Environ Res Public Health.

