# Supplementary material for: General practitioners’ capability, opportunity and motivation to discuss physical activity at the 6-8 week postnatal check in the UK: a qualitative study using the COM-B model
Source: BMJ Open. 2026 Oct 23;16(10):e122011. doi: 10.1136/bmjopen-2026-122011 (PMC13711559; doi:10.1136/bmjopen-2026-122011)
Supplement: Supplementary file 1 [file bmjopen-2026-122011.reviewer_comments.pdf]

PEER REVIEW HISTORY

BMJ Open publishes all reviews undertaken for accepted manuscripts. Reviewers are asked to complete a checklist review form and are provided with free text boxes to elaborate on their assessment. These free text comments are reproduced below.

ARTICLE DETAILS

Title (Provisional)

General practitioners’ capability, opportunity and motivation to discuss physical activity at the 6–8-week postnatal check in the UK: a qualitative study using the COM-B model

Authors

Morwood, Joseph; Rouse, Peter C.; De Vivo, Marlize

VERSION 1 - REVIEW

**Reviewer**	**1**
**Name**	**Delicate, Amy**
**Affiliation**	**University of Birmingham, Applied Health Research**
**Date**	**26-May-2026**
**COI**	**None**

The study is interesting and has the potential to add to the literature around GP 6-8 weeks checks and most importantly enhancing their effectiveness for postnatal women and their families. I have some minor comments for the introduction and discussion. However, I have major comments for the methods and results which I feel need to be addressed before the paper can be published.

Introduction: This could be improved by giving some context to the NHS for non UK readers. What is the NHS, what is a 6-8 week GP check, who has them, how does it fit with wider maternity care.

Methods: These are too brief and would not be able to be replicated. In particular, there is no sense of how the analysis was coducted, by whom, how rigour was assured (the six stages are just listed). There should also be a clear reflexivity statement.

Furthermore:

- Was the analysis iterative (if not, how was information power 'calculated').

- Was there a consent process, what information did potential participants receive about the research (e.g. PIL).

- Were Zoom transcriptions checked for accuracy and by whom? Where they annoymised? Were they returned to participants to be checked?

- Was software used to conduct the analysis?

Results:

The number and demographics of participants should be reported in results not the methods. Do you have any other information about the participants such as the nation they work in, years qualified, area of work e.g. rural/urban, deprivation level, ethnicity.

When were the interviews completed? How long were they? Was it just one interview or repeat?

The reporting of themes is brief and closer to topic descriptions that the interpretive nature of reflexive thamtic analysis.

Discussion:

I sense that one researcher completed the analysis and interpretation and this is therefore a limitation.

Not having PPI or service user participation is a limitation.

**Reviewer**	**2**
**Name**	**Piccinini-Vallis, Helena**
**Affiliation**	**Dalhousie University, Family Medicine**
**Date**	**22-Jun-2026**
**COI**	**None**

This is an interesting qualitative study exploring GPs' perspectives on physical activity counselling at the routine 6-month postpartum visit, using the COM-B model as a framework. The study addresses an important area that has implications for curbing postpartum weight retention, addressing preventive health issues and improving quality of life.

There are some major items that I would like to flag:

1. Reflexive thematic analysis develops themes through an iterative, interpretive engagement with the data. You have a pre-existing framework, in which you systematically indexed data against its categories. Unless I am mistaken, this approach is not usually considered "reflexive" thematic analysis but rather deductive thematic analysis. You even state in your abstract that "Deductive coding was informed by the COM-B framework". Please clarify.

2. I am wondering if the eligibility criterion of having delivered “at least one 6-8 week postnatal check within the last 12 months” needs some explanation. I would not consider someone who has only delivered one such check in the last 12 months as being very current - perhaps lacking experience and competence in that area, potentially influencing your results. Perhaps providing your readers with a sense of the participants’ experience with postnatal care would be helpful.

3. Page 8, Line 49: your overview of theme 2 under “Motivation” suggests some overlap between Motivation and Capability. This is part of a bigger question for me, namely why you decided to not stay consistent with the COM-B model’s constructs under each of the 3 domains, i.e., Physical Capability and Psychological Capability, etc. I think that question needs some explanation, perhaps in the methods section. Your decision to stay broad, at a high level, could potentially explain some of the overlap you noticed.

4. Page 10, paragraph starting on line 45: there really needs to be some reference here to the literature on patient-centredness. For example, the literature on the 5As, or the patient-centred clinical method, in which (e.g., in the latter), the clinician explores the patient’s experience of an issue and understands the patient’s context, and is thus able to find common ground to make recommendations that are meaningful to the patient. The manuscript appears to overlook these established approaches to care that are relevant to this discussion. I would thus consider situating this paragraph within a patient-centred framework, because the findings here are not novel (as viewed through a patient-centred lens).

Minor items include a number of typos or items for clarification that perhaps the journal editing staff would notice, but I will list them here:

Typos:

Page 3, Line 10: insert “physical”: “...sufficient physical activity throughout life.”

Page 3, Line 10: typo: “...minimum levels amounts...”

Page 3, Line 12: wording: “...are suggested to reintroduce...”

Page 3, Line 17: typo: “...vigorous intensity activity decrease...”

Page 3, Line 35: physical activity should not be capitalized

Page 3, Line 36: you mean postpartum physical activity promotion, not pregnancy physical activity promotion

Page 3, Line 45: “de Vivo and Mills to this approach...”: which approach? The sentence is not really clear.

Page 4: The research questions should be stated using the plural for the COM domains (i.e., capabilities, opportunities and motivations).

Page 4, Line 29: "Seven GPs participated...": that sentence belongs in the results section, not the methods section.

Page 4, line 44: please explain what is meant by "information power".

Page 5, Line 40: "This uncertainty appeared linked to perceived gaps in training". This statement does not appear to be supported by the quotes – perhaps it should be moved to the discussion.

Page 6, Lines 9 and 34: Similar to #5 above, you have provided statements that reflect your interpretation of the data. These statements arguably belong in the discussion, not the results section.

Page 9, line 27: no need to redefine "...general practitioners' (GPs')..."

Page 9: It may make more sense to reorder the paragraphs in the discussion, under "comparison with existing literature", to match the order of the COM-B model.

Page 10, line 11: the addition of "previously" when describing highly active individuals, would probably make the sentence for accurate: "...particularly following caesarean section or when advising previously highly active individuals."

Page 10, line 52: The title of this section is "Health Inequalities...". Do you mean inequities?

VERSION 1 - AUTHOR RESPONSE

Dr Amy Delicate's recommendations to the author
Introduction: This could be improved by giving some context to the NHS for non UK readers. What is the NHS, what is a 6-8 week GP check, who has them, how does it fit with wider maternity care.

This recommendation has rightly highlighted the important context of the 6–8-week check for international readers outside of the NHS, where similar postnatal consultations might instead be delivered by paediatricians or obstetricians. Further information on the nature of the 6–8-week check has been added to the Introduction section on page 2, lines 23-30 with the NHS England 'What good looks like' guidance as the relevant policy context:

"In England, women are offered a postnatal consultation with a GP between six and eight weeks after birth. Provision of this consultation has formed part of the GP contractual framework since 2020. NHS England guidance recommends a holistic assessment of maternal physical and mental health and identifies physical activity as an area for health promotion during the consultation. The 6–8-week postnatal check therefore provides an opportunity for GPs to discuss physical activity following childbirth."

Methods: These are too brief and would not be able to be replicated. In particular, there is no sense of how the analysis was conducted, by whom, how rigour was assured (the six stages are just listed). There should also be a clear reflexivity statement.

This feedback was a very useful prompt to substantially expand the Analysis section of the methodology on page 4 lines 10-31, better detailing how the analysis was conducted

and by whom. This also provided the necessary impetus to reflect on the style of analysis. The deductive backbone of the themes precludes labelling the process as solely reflexive and the analysis description now aims to better describe the thematic method:
"Data were analysed thematically using a combination of deductive and inductive coding. The COM-B model provided the conceptual framework, with capability, opportunity and motivation used a priori to structure the deductive analysis. Inductive coding was also used to identify additional patterns within participants' accounts. Following familiarisation with the transcripts, the first author (JM) undertook line-by-line coding using Microsoft Excel. Codes were reviewed iteratively and grouped into themes reflecting participants' experiences of discussing physical activity during the 6–8-week postnatal consultation. Within COM-B, the three overarching components are further specified as physical and psychological capability, physical and social opportunity, and reflective and automatic motivation. These six subcomponents informed the coding process, but the final themes were organised within the three overarching components. This approach reflected the study aim of identifying broader behavioural determinants influencing GPs' discussions of physical activity and accommodated areas of overlap across the COM-B subcomponents. All transcripts were coded and analysed by the first author using Microsoft Excel. The supervisory team provided methodological guidance and contributed to reflexive discussion of the developing analysis but did not independently code the data.
Sample size was informed by the concept of information power.23 The study addressed a focused research aim using a professionally homogeneous sample of general practitioners with direct experience of conducting the 6–8-week postnatal consultation in the UK. Interview data were reviewed throughout data collection to consider the richness and relevance of the information generated, and recruitment concluded when the dataset was judged to provide sufficient information to address the study aim."
Nonetheless, as reflexivity has been a consideration throughout data collection, a Reflexivity statement has been included in the methods section on page 4, line 33 to page 5 line 2:
"The interviews, transcription and analysis were undertaken by the first author (JM) as part of an MSc research project. JM has a professional interest in primary care and physical activity promotion, which informed the choice of research topic and facilitated an understanding of the clinical context. Recognising that prior knowledge and experiences could influence data collection and interpretation, a semi-structured interview guide was used to promote consistency across interviews. Throughout analysis, coding decisions and interpretations were continually reviewed against the original data to ensure themes accurately reflected participants' accounts."

Was the analysis iterative (if not, how was information power 'calculated').
In the rewritten analysis section on page 4 lines 10-31, a clearer description of the iterative nature of the analysis has been detailed, alongside factors that allowed assessment of the data's information power to determine sufficiency:
"Codes were reviewed iteratively and grouped into themes reflecting participants' experiences of discussing physical activity during the 6–8-week postnatal consultation."

Was there a consent process, what information did potential participants receive about the research (e.g. PIL).
A statement about the provision of the electronic consent form was provided in the Participants and recruitment section, page 3 line 30-31:
"Participants were provided with a participant information sheet, and informed consent was obtained via an electronic consent form before the interview."

Were Zoom transcriptions checked for accuracy and by whom? Where they anonymised? Were they returned to participants to be checked?

Clarification has been added that the Zoom transcriptions were checked only by the first researcher against the audio recording of the interview, they were anonymised but not returned to participants prior to analysis as has now been added to the Data Collection section page 4, lines 7-8:
"Interviews were conducted via Zoom between November 2024 and April 2025 by the first author (JM), a practising NHS GP. An initial pilot interview with an NHS GP informed development of the semi-structured topic guide, which was structured around the COM-B domains and refined following a second pilot interview (Supplement A). Sessions lasted 40–65 minutes, were audio-recorded and transcribed using Zoom software, then anonymised and checked against the recordings for accuracy. Transcripts were not returned to participants for comment or correction, and no repeat interviews were conducted."

Was software used to conduct the analysis?
The text now specifies that Microsoft Excel was used for the storage and review of codes and transcript references as detailed now in the Analysis section on page 4 lines 13-14:
"Following familiarisation with the transcripts, the first author (JM) undertook line-by-line coding using Microsoft Excel."

The number and demographics of participants should be reported in results not the methods. Do you have any other information about the participants such as the nation they work in, years qualified, area of work e.g. rural/urban, deprivation level, ethnicity.
The number and basic demographics of the participants have now been included in the Results, page 5 lines 11-16. Information about physical activity levels and pregnancy experience was gathered but unfortunately other information such as years qualified, ethnicity and the socio-economic background of their patient population was not formally recorded, although it is referenced to in the body of each participant's interview:
"Of the 12 GPs who initially consented to participate, seven completed an interview within the study period. Six participants were female and one was male, and four had previously experienced pregnancy. Participants were aged 32–56 years, with a mean age of 40.86 years (SD 8.49). Two reported engaging in less than 90 minutes of physical activity per week, while five reported more than 150 minutes per week."

When were the interviews completed? How long were they? Was it just one interview or repeat?
Interviews were conducted between November 2024 and April 2025 with all interviews lasting between 40 and 65 minutes and no repeat interviews required. This information has been added to the Data Collection section on page 4, lines 2-8:
"Interviews were conducted via Zoom between November 2024 and April 2025 by the first author (JM), a practising NHS GP. An initial pilot interview with an NHS GP informed development of the semi-structured topic guide, which was structured around the COM-B domains and refined following a second pilot interview (Supplement A). Sessions lasted 40–65 minutes, were audio-recorded and transcribed using Zoom software, then anonymised and checked against the recordings for accuracy. Transcripts were not returned to participants for comment or correction, and no repeat interviews were conducted."

The reporting of themes is brief and closer to topic descriptions that the interpretive nature of reflexive thematic analysis.
We agree that the original manuscript did not describe the analytical approach with sufficient clarity and that this created inconsistency between the stated methodology and the presentation of the findings. On reviewing the analysis, it was primarily deductive, with the COM-B model providing the conceptual framework and its three overarching

components structuring the analysis, alongside inductive development of themes within this framework. The manuscript has therefore been revised throughout to describe the analytical approach more accurately rather than characterising it as reflexive thematic analysis. The Analysis section has been expanded on page 4, lines 10-31 and the Results section on pages 5–9 has been revised to provide greater analytical depth and more clearly distinguish the themes developed within the COM-B framework from the framework itself.

I sense that one researcher completed the analysis and interpretation and this is therefore a limitation.
The Methods section on page 5, lines 22-25 has been revised to clarify that all coding and analysis were undertaken by the first author, with the supervisory team contributing to methodological and analytic discussion:
"All coding and analysis were undertaken by the first author. The supervisory team provided methodological guidance and contributed to discussion of the developing analysis but did not independently code the data."
The Strengths and limitations section on page 12, lines 1-5 has also been revised to acknowledge the implications of analysis being undertaken by a single researcher:
"Coding and interpretation were undertaken by a single researcher and therefore reflect their situated analytic perspective. Reflexive discussion with the supervisory team was used throughout the analytic process to interrogate developing interpretations. The study did not involve postnatal women in its design; their involvement may have provided additional perspectives to inform development of the interview guide and interpretation of the findings."

Not having PPI or service user participation is a limitation.
We agree that the absence of patient and public involvement is a limitation of the study. As this work was undertaken as part of an MSc research project, opportunities to incorporate formal PPI were limited within the scope and timeframe of the study. This has now been acknowledged explicitly in the manuscript on page 5 line 7, where we state that patients and the public were not involved in the design, conduct or reporting of the study.
"Patients and the public were not involved in the design, conduct or reporting of this study."
The Strengths and Limitations section on page 12 lines 3-5 now acknowledges that involving postnatal women would have strengthened development of the interview guide and interpretation of findings:
The study did not involve postnatal women in its design; their involvement may have provided additional perspectives to inform development of the interview guide and interpretation of the findings."

Dr Helena Piccinini-Vallis's recommendations to the author

Reflexive thematic analysis develops themes through an iterative, interpretive engagement with the data. You have a pre-existing framework, in which you systematically indexed data against its categories. Unless I am mistaken, this approach is not usually considered "reflexive" thematic analysis but rather deductive thematic analysis. You even state in your abstract that "Deductive coding was informed by the COM-B framework". Please clarify.
We agree with the reviewer's interpretation and have revised the manuscript accordingly. The original wording did not clearly distinguish the role of researcher reflexivity from the analytic approach itself. Although reflexivity informed the research process, the analysis was primarily deductive, with the COM-B model providing the

conceptual framework and its overarching components structuring the analysis, alongside inductive coding within that framework. We have therefore removed the description of the analysis as reflexive thematic analysis. The manuscript has been amended to reflect this in the abstract on page 1 lines 9-10:
"Qualitative study using semi-structured interviews, with data analysed thematically using the COM-B model as the conceptual framework."
The methods on page 4 lines 10-13:
"Data were analysed thematically using a combination of deductive and inductive coding. The Capability, COM-B model provided the conceptual framework, with capability, opportunity and motivation used a priori to structure the deductive analysis. Inductive coding was also used to identify additional patterns within participants' accounts."
A separate reflexivity statement has been retained to describe the researcher's position and consideration of its potential influence on data collection and interpretation, rather than to characterise the analytic method. This is reflected on page 4 line 36 to page 5 line 2:
"Recognising that prior knowledge and experiences could influence data collection and interpretation, a semi-structured interview guide was used to promote consistency across interviews. Throughout analysis, coding decisions and interpretations were continually reviewed against the original data to ensure themes accurately reflected participants' accounts."

I am wondering if the eligibility criterion of having delivered "at least one 6-8 week postnatal check within the last 12 months" needs some explanation. I would not consider someone who has only delivered one such check in the last 12 months as being very current - perhaps lacking experience and competence in that area, potentially influencing your results. Perhaps providing your readers with a sense of the participants' experience with postnatal care would be helpful.
We agree that requiring experience of only one 6–8-week postnatal check within the previous 12 months could include GPs with relatively limited recent experience. However, this criterion was intentionally broad. The study aimed to explore the capability, opportunity and motivation of GPs who may be expected to undertake the 6–8-week postnatal check in routine practice, where frequency of delivery and experience vary between practitioners. Requiring recent experience of at least one consultation ensured that participants could reflect on current practice without restricting recruitment to GPs who deliver postnatal care frequently or have a particular interest in this area. The Introduction on page 2 lines 23-30 has been revised to provide greater context on the role of the 6–8-week postnatal check within general practice and the expectations placed on GPs in delivering this consultation.
"In England, women are offered a postnatal consultation with a GP between six and eight weeks after birth. Provision of this consultation has formed part of the GP contractual framework since 2020. NHS England guidance recommends a holistic assessment of maternal physical and mental health and identifies physical activity as an area for health promotion during the consultation. The 6–8-week postnatal check therefore provides an opportunity for GPs to discuss physical activity following childbirth."

Page 8, Line 49: your overview of theme 2 under "Motivation" suggests some overlap between Motivation and Capability. This is part of a bigger question for me, namely why you decided to not stay consistent with the COM-B model's constructs under each of the 3 domains, i.e., Physical Capability and Psychological Capability, etc. I think that question needs some explanation, perhaps in the methods section. Your decision to stay broad, at a high level, could potentially explain some of the overlap you noticed.
We agree that the broader use of the COM-B framework may have contributed to some overlap between constructs and that this required clearer explanation. The Methods

section has therefore been revised to clarify how the COM-B components were used within the analysis.
The revised text now explains that, while the six COM-B subcomponents informed the coding process, the final themes were organised within the three overarching components of capability, opportunity and motivation. This reflected the study aim of identifying broader determinants influencing GPs' discussions of physical activity and allowed experiences that related to more than one subcomponent to be represented without forcing them into discrete categories.
The Methods section on page 5, lines 17-22 now states:
"Within COM-B, the three overarching components are further specified as physical and psychological capability, physical and social opportunity, and reflective and automatic motivation. These six subcomponents informed the coding process, but the final themes were organised within the three overarching components. This approach reflected the study aim of identifying broader behavioural determinants influencing GPs' discussions of physical activity and accommodated areas of overlap across the COM-B subcomponents."

Page 10, paragraph starting on line 45: there really needs to be some reference here to the literature on patient-centredness. For example, the literature on the 5As, or the patient-centred clinical method, in which (e.g., in the latter), the clinician explores the patient's experience of an issue and understands the patient's context, and is thus able to find common ground to make recommendations that are meaningful to the patient. The manuscript appears to overlook these established approaches to care that are relevant to this discussion. I would thus consider situating this paragraph within a patient-centred framework, because the findings here are not novel (as viewed through a patient-centred lens).
The Discussion section on pages 10, lines 36-41 has been amended to explicitly frame these findings in relation to patient-centred care with references to the patient-centred clinical method and the 5As approach provided on pages 14 lines 28-32. The interpretation of novelty has also been moderated by describing maternal readiness as an important finding rather than presenting patient-centred tailoring itself as a novel clinical concept:
"The concept of pregnancy and the postnatal period as a "teachable moment" for behaviour change may therefore require refinement. While motivation for behaviour change may exist, the findings suggest that timing and delivery are critical, and that receptiveness may vary considerably between individuals. These findings are consistent with established principles of patient-centred care, which emphasise understanding patient's experiences, concerns and priorities before jointly agreeing management plans."

Minor items include a number of typos or items for clarification that perhaps the journal editing staff would notice, but I will list them here:

Page 3, Line 10: insert "physical": "...sufficient physical activity throughout life."
The wording has been corrected in the introduction page 2, lines 7-9
"Both children and adults gain significant reductions in all-cause mortality by engaging in sufficient physical activity throughout life."

Page 3, Line 10: typo: "...minimum levels amounts..."
The wording has been corrected in the introduction page 2 line 9.

Page 3, Line 12: wording: "...are suggested to reintroduce..."
The wording has been corrected in the introduction page 2 lines 11-13.

Page 3, Line 17: typo: “...vigorous intensity activity decrease...”
This wording has been corrected in the introduction page 2 line 14-15.

Page 3, Line 35: physical activity should not be capitalized
This capitalisation has been corrected in the introduction page 2 line 31.

Page 3, Line 36: you mean postpartum physical activity promotion, not pregnancy physical activity promotion
The wording has been revised to refer to postnatal physical activity promotion in the introduction page 2 lines 32-33:
“Resultantly, clinicians’ beliefs about their role in postnatal physical activity promotion has been identified as a research priority.”

Page 3, Line 45: “de Vivo and Mills to this approach...”: which approach? The sentence is not really clear.
This sentence has been clarified in the introduction section page 3 line 1-4 so that the reference to De Vivo and Mills explicitly describes their qualitative approach to exploring healthcare professionals' experiences of providing physical activity advice:
“De Vivo and Mills sought this qualitative insight among NHS Midwives whose perspectives on providing physical activity advice highlighted institutional barriers in lack of resources and training but also generated focus on opportunities to discuss physical activity.”

Page 4: The research questions should be stated using the plural for the COM domains (i.e., capabilities, opportunities and motivations).
The research questions have been revised to use the plural terminology consistently in the introduction page 3 lines 14-19:
“What are GPs’ perceived capabilities to provide physical activity advice and guidance at the 6-8-week postnatal check?
What are GPs’ perceived opportunities to provide physical activity advice and guidance at the 6-8-week postnatal check?
What are GPs’ perceived motivations to provide physical activity advice and guidance at the 6-8-week postnatal check?”

Page 4, Line 29: “Seven GPs participated...”: that sentence belongs in the results section, not the methods section.
The participant count has been retained in the Results section on page 5 lines 11-13.

Page 4, line 44: please explain what is meant by “information power”.
The Methods section has been expanded on page 4 lines 25-31 to explain that information power refers to the adequacy of the dataset for addressing the study aim, and the specific factors used to judge sufficiency have been described: the narrow research aim, professionally homogeneous sample, direct participant experience and richness/relevance of the interview data:
“Sample size was informed by the concept of information power. The study addressed a focused research aim using a professionally homogeneous sample of general practitioners with direct experience of conducting the 6–8-week postnatal consultation in the UK. Interview data were reviewed throughout data collection to consider the richness and relevance of the information generated, and recruitment concluded when the dataset was judged to provide sufficient information to address the study aim.”

Page 5, Line 40: “This uncertainty appeared linked to perceived gaps in training”. This statement does not appear to be supported by the quotes – perhaps it should be moved

to the discussion.
On reflection this statement is not supported by the quotes provided and has been removed from the results section.

Page 6, Lines 9 and 34: Similar to #5 above, you have provided statements that reflect your interpretation of the data. These statements arguably belong in the discussion, not the results section.
These statements have been removed from the results section.

Page 9, line 27: no need to redefine "...general practitioners' (GPs')..."
This redefinition has been removed from the discussion.

Page 9: It may make more sense to reorder the paragraphs in the discussion, under "comparison with existing literature", to match the order of the COM-B model.
The ordering of the Discussion has been reviewed and the current structure retained. This retained order enables the narrative to move from the broadest and most consistent finding towards more specific considerations. Although this does not follow the COM-B domains in sequence, it was felt this order provided a more clinically meaningful discussion of the findings. We have therefore not reordered this section, but have aimed to ensure that the capability, opportunity and motivation findings remain clearly represented throughout.

Page 10, line 11: the addition of "previously" when describing highly active individuals, would probably make the sentence for accurate: "...particularly following caesarean section or when advising previously highly active individuals."
This sentence on page 10 line 11-13 has been clarified to highlight that these patients were previously active.

Page 10, line 52: The title of this section is "Health Inequalities...". Do you mean inequities?
This is a valid query, however the use of inequalities has been retained to reflect the terminology in the cited literature and framing of the findings.

VERSION 2 - REVIEW

**Reviewer**	**1**
**Name**	**Delicate, Amy**
**Affiliation**	**University of Birmingham, Applied Health Research**
**Date**	**16-Sep-2026**
**COI**	

Congratulations on the paper. I feel the amendments made to address comments from myself and Dr Piccinini-Vallis have enhanced the paper which I enjoyed reading.

**Reviewer**	**2**
**Name**	**Piccinini-Vallis, Helena**

**Affiliation**	**Dalhousie University, Family Medicine**
**Date**	**24-Sep-2026**
**COI**	

All my questions have been addressed. I have no further concerns.
