# Supplementary material for: General practitioners’ capability, opportunity and motivation to discuss physical activity at the 6-8 week postnatal check in the UK: a qualitative study using the COM-B model
Source: BMJ Open. 2026 Oct 23;16(10):e122011. doi: 10.1136/bmjopen-2026-122011 (PMC13711559; doi:10.1136/bmjopen-2026-122011)
Supplement: Supplementary file 2 [file bmjopen-16-10-s001.docx]

**Supplement A**

**Semi-Structured Interview Guide**

|  |  | **Global Question** | **Global Follow up Questions** |
| --- | --- | --- | --- |
|  | **General** | - *When you deliver a 6-8-week check, what are your main priorities?* | - *What determines your priorities do you think?* |
|  |  | **Core Question** | **Follow up Questions** |
| **Perceived Capability** | **Physical** | - *Can you tell me about a time you have ever discussed Physical Activity at the 6-8 week check?* | - *If no – why not* - *If yes – can you tell me more about how you were able to?* |
|  |  | - *What sort of information do you know about what and how Physical Activity is recommended following pregnancy?* | - *Can you tell me much about what types of Physical activity are recommended?* - *What sources of information have you used?* - *Where did you get this information?* - *Was it provided proactively or not?* |
|  | **Psychological** | - *If you were talking to a mother at the 6-8 week check, how confident would you feel discussing physical activity with them?* | - *Can you explain why not if so?* - *What are the barriers to you feeling confident?* - *Are there certain topics you feel you lack confidence in?* - *Is there anything that would make you feel more confident?* |
| **Perceived Opportunity** | **Social** | - *Think about your workplace + colleagues – how do they support you to talk about Physical Activity?* | - *Would you describe your colleagues as active?* - *Do you know if your Practice is an ‘Active Practice?’* |
|  |  | - *Are there ways in which your workplace or colleagues make it difficult to talk about physical activity?* |  |
|  | **Physical** | - *What sort of resources would you need to make it easier to discuss physical activity at the 6-8 week check?* | - *How much time do you have to discuss PA during the 6-8 week check? Enough?* - *Do you have any local physical activity resources available for postpartum women?* |
| **Perceived Motivation** | **Reflective** | - *What do you think are the benefits to women being counselled by their GP on activity at the 6-8 week check?* | - *Has personal experience of pregnancy influenced this motivation? Are there previous patient experiences that influence this motivation?* |
|  |  | - *What do you think are the disadvantages to women being counselled by their GP on activity?* |  |
|  | **Automatic** | - *Do you find anything motivating or rewarding to discuss PA with postpartum women?* |  |
